# Motion Planning and Control with Environmental Uncertainties for Humanoid Robot

**DOI:** 10.3390/s24237652

**Published:** 2024-11-29

**Authors:** Zhiyong Jiang, Yu Wang, Siyu Wang, Sheng Bi, Jiangcheng Chen

**Affiliations:** 1 Robotics Engineering Center, The 21st Research Institute, China Electronics Technology Group Corporation, Shanghai 200233, China; jiangzy2013@163.com; 2 Shenzhen Academy of Robotics, Shenzhen 518057, China; siyu_wang@szarobots.com (S.W.); bisheng@szarobots.com (S.B.); chenjiangcheng@szarobots.com (J.C.)

**Keywords:** humanoid robots, motion planning, dynamic balance, perceptive control, environment uncertainties

## Abstract

Humanoid robots are typically designed for static environments, but real-world applications demand robust performance under dynamic, uncertain conditions. This paper introduces a perceptive motion planning and control algorithm that enables humanoid robots to navigate and operate effectively in environments with unpredictable kinematic and dynamic disturbances. The proposed algorithm ensures synchronized multi-limb motion while maintaining dynamic balance, utilizing real-time feedback from force, torque, and inertia sensors. Experimental results demonstrate the algorithm’s adaptability and robustness in handling complex tasks, including walking on uneven terrain and responding to external disturbances. These findings highlight the potential of perceptive motion planning in enhancing the versatility and resilience of humanoid robots in uncertain environments. The results have potential applications in search-and-rescue missions, healthcare robotics, and industrial automation, where robots operate in unpredictable or dynamic conditions.

## 1. Introduction

Humanoid robots are typically designed and work for static environments, but real-world applications demand robust performance under dynamic, uncertain conditions. Many significant studies and achievements have been made in recent years based on some well-known hardware platforms, such as HRP series humanoid robots [1,2,3], Atlas [4], Cassie [5], and Digit [6]. HRP-2 humanoid robot which has 1540 mm height, 58 kg weight and 30 degrees of the freedom, is one of the earliest series of platforms for researchers to implement their algorithms. Atlas is a highly advanced humanoid robot developed by Boston Dynamics, known for its impressive agility, balance, and ability to perform complex movements such as running, jumping, and navigating challenging environments. Cassis and Digit are humanoid robots designed for advanced mobility and task execution, with Cassis specializing in human-robot interaction and Digit focusing on delivery and logistics tasks with exceptional agility and dexterity. Many advanced control algorithms have been applied in these excellent platforms to draw maximum performance. These algorithms aim to achieve a more responsive and agile motion by improving the dynamic balance of the robot or manipulated objects.

Path planning, a critical aspect of humanoid robot control, has seen the integration of various intelligent techniques. For instance, neural network-based approaches have shown promise in optimizing robotic paths. Cellular Neural Networks (CNNs) have been utilized for real-time, collision-free path generation, leveraging analogies with harmonic functions to ensure smooth and dynamic trajectories in complex environments [7,8]. Other works, such as crossover recombination-based optimization algorithms, effectively address multi-constraint optimization problems like UAV path planning by enhancing path smoothness and adaptability in three-dimensional spaces [9]. Additionally, methods tailored for specific contexts, such as path planning for needle insertion in deformable soft tissues, illustrate the broader applicability of path planning techniques across diverse domains [10,11].

Static balance is easier and safer, as the system remains stable even if all joints freeze. Kajita [12] introduced a seminal method for achieving stable walking in humanoid robots using Zero Moment Point (ZMP) control combined with preview control. This approach leverages the preview gain, which is determined by solving the Riccati equation [13]. Achieving dynamic balance with uncertainties in robots presents two main challenges. First, humanoid robots or manipulated objects may be underactuated, meaning not all poses, velocities, or accelerations are feasible due to limited contact points and friction constraints [14]. Second, the system must adapt to dynamic uncertainties from both internal factors, like model errors and communication delays, and external factors, such as slippery surfaces or unexpected forces [15]. The integration of advanced filters, such as the Model Predictive-Based Unscented Kalman Filter (MP-UKF), has demonstrated improved adaptiveness in nonlinear and high-dynamic systems. This approach compensates for model errors in real time, enhancing the performance of navigation systems in environments with high maneuverability demands [16].

Traditional methods are generally relying on offline motion planning and linear control, which is inadequate for fully exploiting hardware capabilities and responding effectively to dynamically changing environments. In contrast, Model Predictive Control (MPC) offers trajectory generation in the nonlinear domain, enabling the optimal use of the robot’s kinematic range and torque limits [17]. Furthermore, frequent motion replanning based on the robot’s current state enhances robustness against disturbances. As a result, the application of MPC in legged robotic systems has been a growing area of research over the past decade. In ICRA 2022, there are 19 out of 28 workshop papers related to MPC [17]. In the early stages of legged robotics research, MPC was introduced for the online generation of walking patterns in bipedal and humanoid robots, aiming to enhance responsiveness to disturbances and user inputs. Here, the term online generation means that the trajectory keeps updating while the robot is moving.

There are many different types of MPC algorithms. In general, MPC uses the model of the system under control to predict its behavior in a defined prediction horizon [18]. Following that, the objectives of the system are defined as a cost function and the control problem becomes minimizing the errors between the model predicted values and reference values. In addition to dynamic modeling-based control approaches, reinforcement learning has emerged as a compelling alternative for controlling humanoid robots. Leveraging tools like Isaac Sim and Gym provided by NVIDIA, researchers from ETH Zurich and NVIDIA have demonstrated a novel and promising method for controlling various types of legged robots [19]. By training numerous robots in parallel within a high-fidelity physics simulation environment, these systems achieve real-world locomotion after addressing key sim-to-real challenges.

In addition, there are always unpredictable factors in a humanoid robot’s operating environment, which we can refer to as environment uncertainties. These uncertainties include variations in friction, changes in terrain slope, and unpredictability in object poses or external disturbances. Significant research efforts have been devoted to addressing uncertainties in control systems, including approaches such as uncertain iterative optimal attitude control [20] and uncertain optimal design strategies [21]. However, these uncertainties remain a substantial challenge, especially for robots performing tasks such as manipulation and locomotion. Such tasks require adaptive strategies to effectively respond to dynamic changes in the environment. While traditional Model Predictive Control (MPC) can handle certain types of environmental uncertainties to ensure stable walking, its capabilities are limited when faced with more complex or unpredictable scenarios.

MPC in humanoid robots, while effective for planning and optimization, has several drawbacks. First, MPC is computationally intensive due to its reliance on solving optimization problems at every time step, which can lead to latency and limit real-time performance, especially in highly dynamic environments [22]. This computational burden increases with the complexity of the robot’s model and the number of constraints considered. Additionally, MPC requires accurate models of the robot’s dynamics [17], which can be challenging to obtain due to uncertainties or approximations in the robot’s mechanical structure and external forces like friction or disturbances. Another issue is that MPC often struggles with nonlinearity in humanoid movements [23], and achieving fast responses or agile behaviors may require more specialized or approximated control strategies. Lastly, tuning MPC parameters can be difficult, requiring extensive trial and error to balance control performance and computational efficiency [24].

In this paper, a perceptive motion reference system for humanoid robots operating in dynamic and uncertain environments is developed. It is designed to improve dynamic balancing and motion synchronization. Instead of relying on traditional time-based reference tracking, this method shifts to a path-following approach guided by real-time sensory information, especially force, torque, and inertia data. The perceptive reference is derived from dynamic balance criteria such as the Zero Moment Point (ZMP). Compared to traditional MPC algorithms, the key difference lies in its adaptability: when disturbances occur, the system temporarily pauses the motion and re-synchronizes the movements without requiring complete trajectory re-planning, providing robustness and adaptability. This method is particularly suited to underactuated systems where perfect control is not feasible, and external disturbances (like soft ground or object blocking) are common. The rest of this paper is organized as follows: Section 2 details the perceptive motion reference framework, focusing on how it addresses dynamic balance and environmental uncertainties in multi-limb robotic systems. Section 3 presents the trajectory planning methodology, illustrating the synchronization of legs and the Center of Gravity (CoG) using the proposed perceptive approach. Section 4 provides simulation and experimental results, demonstrating the framework’s robustness in handling disturbances and unexpected conditions, and finally, Section 5 concludes the paper with a summary.

## 2. Perceptive Motion Reference Framework

### 2.1. System Overview

**Figure 1:**
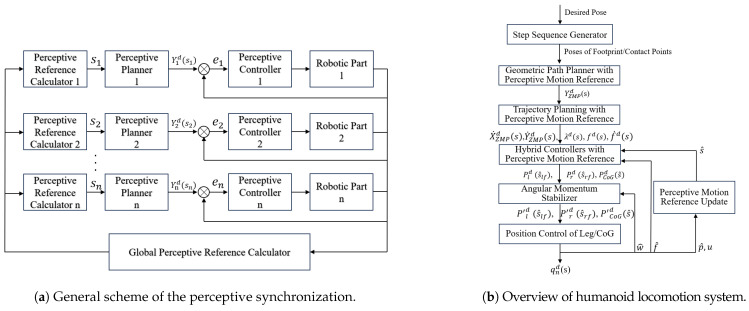
Locomotion system structure.

Figure 1a illustrates the overall perceptive planning and control framework. The central concept is the use of a perceptive motion reference, denoted as a scalar *s* representing the system’s state derived from sensory data. This reference is used to plan the desired motion trajectory of subsystems, such as limbs based on the value of *s*. The system operates in a closed loop by continuously computing *s* in real time and determining the required motion, such as the desired pose of the *i*-th limb, represented as $Y_{i}^{d}\left(s\right)$, according to the perceptive plan. The control error, like the error for the *i*-th limb, $e_{i}\left(s\right)$, is also calculated based on this reference. The direct relationship between the perceptive reference and the system state aids in achieving coordinated synchronization. This integration means that planning is now part of the real-time control loop.

In humanoid locomotion, this system enables the robot to intuitively swing its leg when the Zero Moment Point (ZMP) shifts to specific areas, ensuring dynamic balance, while also minimizing the L2-norm error of the ZMP. Figure 1b shows the overall locomotion system and intermediate parameters, where all the desired values, including force, orientation, and speed, are functions of the perceptive motion reference *s*. This flexibility helps set more reasonable desired values when the assumptions made during planning, such as timing or spatial constraints, are not met. For instance, trajectory generation algorithms [25] may assume an instant transition between the single support phase (SSP) and double support phase (DSP), but foot landing can be affected by various unpredictable factors like contact point irregularities, causing deviations in planned trajectories. Our controllers adjust the desired values according to the current perceptive motion reference, which account for the impact of any invalid planning assumptions.

**Figure 2:**
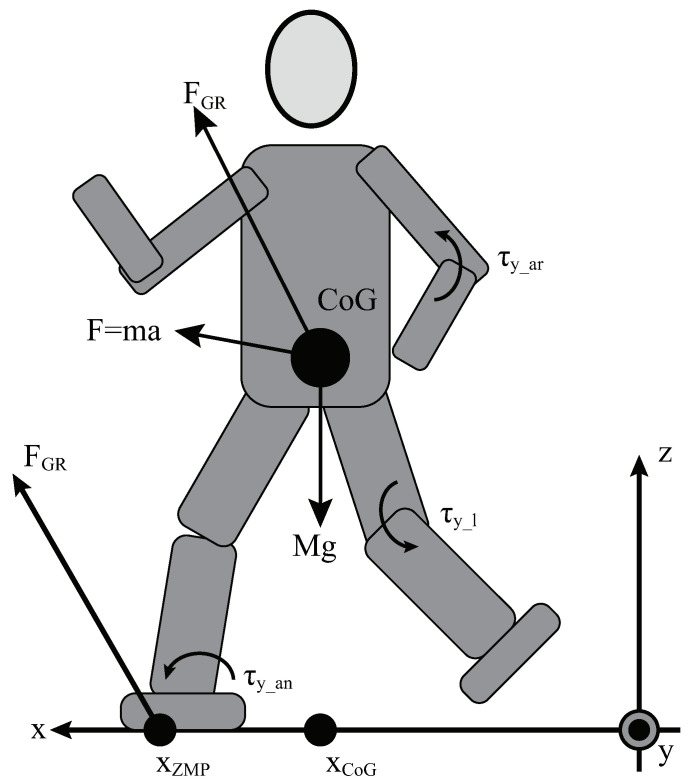
A simple illustration of a dynamic model for a humanoid robot.

Figure 2 is a 3D linear inverted pendulum model of a humanoid robot. To maintain a consistently low spin angular momentum, similar to human walking on flat ground, a whole-body angular stabilizer uses torque from arm swings and ankle rotations (denoted as $\tau_{y\_ar}$ and $\tau_{y\_an}$ which are illustrated in Figure 2) to counterbalance the additional angular momentum [26] caused by leg swings (as $\tau_{y}\_l$ in Figure 2). This stabilization minimizes the deviation of the Center of Gravity (CoG). The process introduces the centroidal moment pivot (CMP), a key ground reference through which the shifted ground reaction force (FGR) can pass through the CoG. The CMP is controlled to align with the ZMP when the horizontal component of angular momentum around the CoG is neutralized. For the sake of brevity, the specific details of this control are not elaborated in the paper. However, this approach allows us to assume that the net moment around the CoG is zero. In flat ground walking tasks, the desired angular position $\theta_{d}\left(s\right)$ is set to a constant value of zero.

### 2.2. Design of the Perceptive Motion Reference

As discussed earlier, the perceptive reference *s* replaces time as the motion reference for planning and synchronizing the movements of both the legs and the Center of Gravity (CoG). Unlike traditional path-following methods, various studies [27,28] suggest that this non-time-based motion reference should incorporate the following: First, the system’s inherent characteristics, including its kinematics, dynamics, and transfer functions, which are closely linked to the system states that are critical for the task; second, the key objective or essential criterion of the task, such as controlled values like distance traveled, tangential speed, etc.

In our locomotion framework, we adopt the Zero Moment Point (ZMP) balance criterion [25], where the ZMP must remain within the center of the support polygon at all times during walking. The ZMP’s position is connected to the CoG through a linear inverted pendulum dynamic model and is constrained by the variable support polygon created by the feet. Therefore, the distance traveled by the ZMP along the planned reference path is chosen as the perceptive motion reference *s*. For a given geometric reference path of the ZMP on a 2D xy-plane, it can be expressed as piecewise functions $y=f_{i}^{\prime }\left(x\left(s\right)\right)=f_{i}\left(s\right)\in R$, where i∈R and i=1,…,m (*m* being the number of ZMP path segments), and $s\in [0,s_{end}]$. The scalar parameter *s* is calculated based on the real-time projected point $P_{p}\left(x_{p},y_{p}\right)$ of the current ZMP position $P_{ZMP}\left(x_{ZMP},y_{ZMP}\right)$ onto the specified ZMP geometric reference path. The ZMP’s traveled distance along the reference path, as calculated in Equation (1), represents the support point of the 3D inverted pendulum system. This distance contains vital dynamic information that can continuously guide the movement of other body parts. Similarly to time, the ZMP traveled distance is a one-dimensional parameter, which makes it convenient to use as a motion reference for parameterizing the body’s trajectories.

(1) $$s=\sum_{i=1}^{m}\int_{x_{i}}^{x_{i}+1}\sqrt{1+{\left(f_{i}^{\prime }\left(x\right)\right)}^{2}}dx+\int_{x_{m}}^{x_{p}}\sqrt{1+{\left(f_{i}^{\prime }\left(x\right)\right)}^{2}}dx$$

Accordingly, the perceptive motion reference for a foot (denoted by $s_{lf}$ for the left foot or $s_{rf}$ for the right foot) is defined by the distance traveled along its reference path, starting from the initial swing point to the current projected position of the left or right foot on that path. These reference values are computed based on the current value of *s* to ensure balance, as will be explained in the following section.

### 2.3. Perceptive Motion Spatial Planning

The 3D LIP model in Figure 2 can describe the system dynamics and relationship between the CoG and ZMP as the following equation ($F_{x}$, $F_{y}$ and $F_{z}$ are the three direction components of ground reaction force *F*):

(2) $$\begin{array}{c} x_{ZMP}=x_{CoG}-\frac{F_{x}}{F_{z}+Mg}z_{CoG}-\frac{\tau_{y}\left({\vec{r}}_{CoG}\right)}{F_{z}+Mg}\\ y_{ZMP}=y_{CoG}-\frac{F_{y}}{F_{z}+Mg}z_{CoG}+\frac{\tau_{x}\left({\vec{r}}_{CoG}\right)}{F_{z}+Mg} \end{array}$$

Equation (2) also encapsulates the dynamics of scenarios involving manipulated objects with a significant volume, where a single contact point exists with a robotic gripper or finger on the object’s surface. Similarly, it applies to floating space manipulators interacting with objects of a comparatively larger mass. Consequently, the subsequent analysis can be generalized to encompass specific cases of dexterous manipulation and space-based robotic systems.

**Figure 3:**
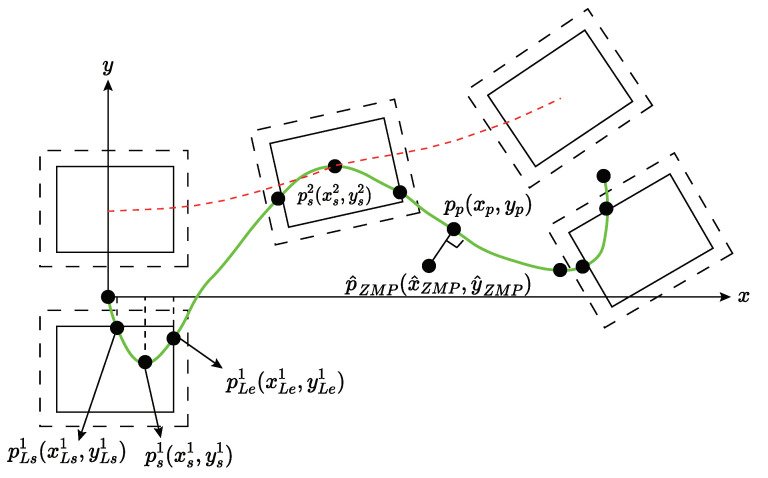
A geometric illustration of reference path planning.

In humanoid locomotion, Figure 3 shows the ZMP reference path (green curves) and the left foot’s path (red-dashed line) on the x–y plane. Dotted squares represent actual footprints, and solid squares mark valid support polygons to prevent ZMP from reaching the footplate edges and tilting the robot. The origin is set between the feet at the task’s start. For each leg swing, the ZMP path must stay within the support leg’s valid polygon to maintain balance, linking leg motion references ($s_{lf}$, $s_{rf}$) to the main reference *s* as described in Equation (3), where *i* is the step number. $s_{Le}^{i}$ and $s_{Ls}^{i}$ correspond to the main reference *s* end and start values for the *i*-th left foot swing, while $s_{lf_{e}}^{i}$ and $s_{lf_{s}}^{i}$ indicate the start and end values of the left foot’s motion reference $s_{lf}$ for that step.

(3) $$s_{lf}=s_{lf_{s}}^{i}+\left(s_{lf_{e}}^{i}-s_{lf_{s}}^{i}\right)\cdot \frac{\min\left(s,s_{Le}^{i}\right)-s_{Ls}^{i}}{s_{Le}^{i}-s_{Ls}^{i}}$$

With Equation (3), it is very easy to know the first left foot swing based on Figure 3. For the other steps, we can see a similar relationship. Unlike [29], our method uses the ZMP to handle early or late contact and respond to foot blocking in the air.

### 2.4. Perceptive Motion Temporal Planning

Our primary consideration is dynamic balance, which requires the ZMP controlled by CoG motion to be located in the support polygon formed by one’s foot. This requires us to develop a foot trajectory generator that initiates the swing when the LIP and walking direction align, and design a ZMP tracking system to ensure the CoG trajectory keeps the ZMP on its reference path. According to the perceptive reference, a third-order polynomial trajectory of the ZMP and feet is planned for the smoothness of the trajectory. The boundary conditions are set based on the desired forward walking speed $v_{x}^{d}$, determined by either user input or the perception module. Using these conditions, two segments of the ZMP trajectories can be derived as Equation (4).

(4) $$\begin{array}{c} {\dot{x}}_{ZMP}^{d}\left(s=s_{L}^{1}e\right)=v_{x}^{d}\\ {\dot{x}}_{Z}^{d}MP\left(s=s_{R}^{2}e\right)=v_{x}^{d}\\ {\dot{x}}_{ZMP}^{d}\left(s=s_{s}^{2}\right)=0.0 \end{array}$$

Based on this condition, three pieces of the right foot trajectory in 3D $v_{rf}^{d}=\left({\dot{x}}_{rf}^{d},{\dot{y}}_{rf}^{d},z_{rf}^{d}\right)$ can be obtained with the boundary conditions. It is quite similar for other steps. Generally, as mentioned in [7], the ZMP preview controller performs much better than a linear-quadratic optimal controller if it considers the future reference position and minimizes ZMP errors accordingly. We define the LIP input variable u as the temporal second derivative of the acceleration of the CoG:

(5) $$u=\left\{\begin{array}{cc} \frac{d}{dt}{\ddot{x}}_{CoG}=u_{x} & \mathrm{In}\;\mathrm{sagittal}\;\mathrm{plane}\\ \frac{d}{dt}{\ddot{y}}_{CoG}=u_{y} & \mathrm{In}\;\mathrm{frontal}\;\mathrm{plane} \end{array}\right.$$

The ZMP equation can be translated into a discretized dynamical system [10] with the control cycle duration T. And, the mentioned ZMP future reference position can be obtained as:

(6) $$p^{ref}\left(k\right)=\left\{\begin{array}{c} x_{ZMP}^{d}\left(kT\right)=x_{p}+kT*{\dot{\gamma }}^{d}\left(s\right)\\ y_{ZMP}^{d}\left(kT\right)=f_{i}\left(x_{ZMP}^{d}\left(kT\right)\right) \end{array}\right.$$

Equation (6) is derived from a discretized dynamical system [30] with the control cycle duration *T*, and *k* is the step. Based on Equation (6), we can have the optimal input of the minimized performance index in a $N_{p}$ sized preview window, and it can be described with:

(7) $$J=\sum_{i=k}^{N_{p}}Q_{e}e{\left(i\right)}^{2}+\Delta x^{T}\left(i\right)Q_{x}\Delta x\left(i\right)+R\Delta u^{2}\left(i\right)$$

To optimize, we can use an online solver. The optimal controller that minimizes *J* is:

(8) $$u\left(k\right)=-G_{i}\sum_{i=0}^{k}e\left(k\right)-G_{x}x\left(k\right)-\sum_{j=1}^{N_{p}}G_{p}\left(j\right)p^{ref}\left(k+j\right)$$

The gain values $G_{x}$, $G_{x}$ and $G_{p}$ can be derived from [30].

## 3. Perceptive Motion Control

### 3.1. Dynamics Model

The full dynamics model of the humanoid robot is established in [31]:

(9) $$H\left(q\right)\ddot{q}+C\left(q,\dot{q}\right)=B\left(q,\dot{q}\right)\tau +\Psi \left(q\right)F$$

where H(q) is the system inertia matrix, $C(q,\ddot{q})$ represents the gravity and Coriolis forces, $B(q,\dot{q})$ is the control input map, and Ψ(q) is a transformation matrix, converting external forces *F* into the corresponding generalized forces acting on the system. Here, we defined the point on the external surface where a shifted ground reaction force vector F would pass through and go across the CoG as Centroid Moment Pivot (CMP). The constraint equation can be described as:

(10) $$\left({\vec{r}}_{CMP}-{\vec{r}}_{CoG}\right)\times \vec{F}=0\;\mathrm{and}\;z_{CMP}=0$$

Hence, we can have a conversion from ZMP to CMP, and it can be described as:

(11) $$\begin{array}{c} x_{CMP}=x_{ZMP}+\frac{\tau_{y}\left({\vec{r}}_{CoG}\right)}{F_{z}+Mg}\\ y_{CMP}=y_{ZMP}+\frac{\tau_{x}\left({\vec{r}}_{CoG}\right)}{F_{z}+Mg} \end{array}$$

### 3.2. Translational Dynamics Equilibrium Perceptive Model

In the single-support phase, where the only external force is the ground reaction force *F* on the stance foot, the CoG position can be described using forward kinematics as $x_{CoG}\left(q\right)$ and $y_{CoG}\left(q\right)$ in a local coordinate system at the ankle. The translation equilibrium is then expressed as:

(12) $$M{\left[\begin{array}{ccc} \ddot{x} & \ddot{y} & \ddot{z} \end{array}\right]}^{T}+mg{\left[\begin{array}{ccc} 0 & 0 & 1 \end{array}\right]}^{T}={\left[\begin{array}{ccc} F_{x} & F_{y} & F_{z} \end{array}\right]}^{T}$$

In the single-foot stance phase, CoG has accordingly $q^{d}\left(s\right)$ in joint space based on inverse kinematics. Hence, Equation (12) can be expressed as:

(13) $$\begin{array}{c} M\frac{\partial x_{CoG}\left(q\right)}{\partial q}\ddot{q}+M{\dot{q}}^{T}\frac{\partial^{2}x_{CoG}\left(q\right)}{\partial q^{2}}\dot{q}=F_{x}\\ M\frac{\partial y_{CoG}\left(q\right)}{\partial q}\ddot{q}+M{\dot{q}}^{T}\frac{\partial^{2}y_{CoG}\left(q\right)}{\partial q^{2}}\dot{q}=F_{y}\\ M\frac{\partial z_{CoG}\left(q\right)}{\partial q}\ddot{q}+M{\dot{q}}^{T}\frac{\partial^{2}z_{CoG}\left(q\right)}{\partial q^{2}}\dot{q}=F_{z} \end{array}$$

There should be a common constraint for each foot’s friction $\left|F_{Lxy}\right|<\mu F_{Lz}$, $\left|F_{Lz}\right|\geq 0$, $\left|F_{Rxy}\right|<\mu F_{Rz}$, and $\left|F_{Rz}\right|\geq 0$. The friction constraint directly influences whether the desired CoG state is valid and achievable based on the potential ground reaction forces on the feet. And the acceleration relationship can be expressed as $a_{xy}\leq \mu \left(a_{z}+g\right)$ and $a_{z}\geq -g$.

### 3.3. Rotational Dynamics Equilibrium Perceptive Model

With the actuated ankle joint, which connects the foot to the rest of the body, remaining stationary, the torque equilibrium around the joint is maintained as:

(14) $$\tau_{a}=Mgx_{CoG}-p_{x}F_{z}-h_{f}F_{x}$$

where $\tau_{a}$ is the overall torque around the ankle joint and it equals the angular momentum of the robot around the ankle joint ${\dot{\sigma }}_{a}$. According to Equations (13) and (14), the dynamics model in terms of the angular momentum would be expressed as:

(15) $$\left(W_{0}\left(q\right)+p_{x}W_{p}\left(q\right)\right)\ddot{q}+l_{0}\left(q,\dot{q}\right)+p_{x}l_{p}\left(q,\dot{q}\right)=0$$

where:

$$W_{0}\left(q\right)=W\left(q\right)+Mh_{f}\frac{\partial x_{COG}\left(q\right)}{\partial q}$$

$$W_{p}\left(q\right)=M\frac{\partial z_{COG}\left(q\right)}{\partial q}$$

$$l_{0}\left(q,\dot{q}\right)={\dot{q}}^{T}\frac{\partial W(q)}{\partial q}\dot{q}-mgx_{COG}\left(q\right)+mh_{f}{\dot{q}}^{T}\frac{\partial^{2}z_{g}\left(q\right)}{\partial q^{2}}\dot{q}$$

$$l_{p}\left(q,\dot{q}\right)=m{\dot{q}}^{T}\frac{\partial^{2}z_{g}\left(q\right)}{\partial q^{2}}\dot{q}+Mg$$

By shifting the rotation axis from the ankle to the ZMP in the sagittal and frontal planes, the angular momentum $\sigma_{p}$ can be expressed as $\sigma_{p}=\sigma_{a}+px\left(M{\dot{z}}_{CoG}\right)-h_{f}\left(M{\dot{x}}_{CoG}\right)$. Taking the derivative of both sides, the torque around the ZMP, $\tau_{px}$ can be represented as:

(16) $$\begin{array}{c} {\dot{\sigma }}_{px}=\tau_{px}=m{\dot{p}}_{x}{\dot{z}}_{CoG}+Mg\left(x_{CoG}-p_{x}\right)\\ {\dot{\sigma }}_{py}=\tau_{py}=m{\dot{p}}_{y}{\dot{z}}_{CoG}+Mg\left(y_{CoG}-p_{y}\right) \end{array}$$

### 3.4. Impact Dynamics Equilibrium Perceptive Model

An impact model is required to link the analysis across different single-leg support phases. For instance, when the left leg, acting as the swing leg, lands at time $t_{c}$ and becomes the support leg, there is a brief double-leg support phase where the robot’s configuration *q* remains unchanged. The right leg then lifts off and becomes the swing leg. The hybrid controller ensures a smooth, non-bouncing landing, with the vertical velocity of the landing foot set to zero post-impact. However, the horizontal velocity $\dot{q}$ changes, and this is linearly represented by a 6 × 6 matrix I(q). Additionally, the roles of the left and right feet are swapped using a permutation matrix X, allowing the single-leg support analysis to be reused. The impact effect in joint space is detailed in [32] as:

(17) $$\begin{array}{c} q^{+}=X\left(q^{-}\right)\\ {\dot{q}}^{+}=X\left(I\left(q\right){\dot{q}}^{-}\right) \end{array}$$

Here, ${\dot{q}}^{+}$ and ${\dot{q}}^{-}$ represent the velocity vectors just after and just before the impact. To ensure the desired CoG reference path aligns with the impact dynamics described in Equation (17), the final velocity of the i-th step ${\dot{q}}^{d}{\left(1\right)}^{-}$ just before impact must match the initial velocity of the i+1-th step ${\dot{q}}^{d}{\left(0\right)}^{+}$ as shown below:

(18) $$\frac{dq^{d}\left(0\right)}{ds}\dot{(}{s)}_{i+1}\left(0\right)=\mathrm{XI}\left(q^{d}\left(1\right)\right)\frac{dq^{d}\left(1\right)}{ds}{\dot{s}}_{i}\left(1\right)$$

Assuming ${\dot{s}}_{i+1}=\gamma {\dot{s}}_{i}$, where γ>0 is a scalar, and with the foot planner determining $q^{d}\left(1\right)$ and $\frac{dq^{d}\left(1\right)}{ds}$, the equation can be reformulated as a condition for $\frac{dq^{d}\left(0\right)}{ds}$ as:

(19) $$\frac{dq^{d}\left(0\right)}{ds}=\mathrm{XI}\left(q^{d}\left(1\right)\right)\frac{dq^{d}\left(1\right)}{ds}\gamma$$

Hence, a feasible series of $\frac{dq^{d}\left(0\right)}{ds}$ solution is provided.

### 3.5. Perceptive Control Law Formulation

The “hybrid” leg controller operates as a velocity closed-loop during the swing phase and a dynamic model-based closed-loop when the leg is in support. A landing event-triggered switch is used to transition between these two control modes.

For the velocity controller, the reference velocity vector ${\dot{f}}^{d}$ for the left and right foot is provided as $\left[{\dot{x}}_{lf}^{d}\left(s^{★}\right),{\dot{y}}_{lf}^{d}\left(s^{★}\right),{\dot{z}}_{lf}^{d}\left(s^{★}\right)\right]$ or $\left[{\dot{x}}_{rf}^{d}\left(s^{★}\right),{\dot{y}}_{rf}^{d}\left(s^{★}\right),{\dot{z}}_{rf}^{d}\left(s^{★}\right)\right]$, depending on the global perceptive reference $s^{★}\in \left[0,s_{max}^{★}\right]$. The foot speed error is calculated as $e_{f}=R\left({\dot{f}}^{d}-\dot{f}\left(t\right)\right)$, where R is the transformation matrix from world coordinates to the robot’s local frame at the pelvis. The velocity controller is then designed accordingly as:

(20) $${\dot{q}}^{d}=J^{-1}\left(q\right)\left(k_{p}\left(\dot{f}-\dot{f}\left(t\right)\right)+k_{i}\left(f^{d}-f\left(t\right)\right)\right)$$

where, J(q) represents the Jacobian matrix of the swing leg, and $k_{p}$, $k_{i}$ are the parameters for the PI controller. The desired joint velocity ${\dot{q}}^{d}$ is then forwarded to the lower-level joint velocity controllers for execution. To minimize unwanted bouncing and oscillations, the transition from the velocity controller (V) for the swing foot to the force controller (F) for the stance foot is based on a force-related condition. These relationship can be represented with equations:

(21) $$\begin{array}{c} V=(\left({\dot{z}}_{f}<0\wedge F_{z}\left(t_{c}\right)=F_{z,max}\right)\vee F_{z}>F_{z,max})\\ F=(\left({\dot{z}}_{f}>0\wedge F_{z}\left(t_{c}\right)=F_{z,max}\right)\vee F_{z}>F_{z,max}) \end{array}$$

where $F_{z,max}$ represents the expected contact force on a single foot during the double support phase, while $F_{z}$ is the ground reaction force in the z axis, as measured by the 6-axis force–torque sensors on the ankles (providing outputs for forces $F_{x}$, $F_{y}$, $F_{z}$ and torques $\tau_{x}$, $\tau_{y}$, $\tau_{z}$). The switch to the force controller (F) occurs at $t_{c}$ when $F_{z}=F_{z,max}$. Once the switch is made, the stance leg controller, designed with feedforward and feedback mechanisms, ensures the CoG reference path $q^{d}\left(s\right)$ and the ZMP position $p^{d}\left(s\right)$ are achieved. The control inputs are joint torques $\tau_{j}$ (for j=1,…,6) and the reference velocity $\dot{s}$, where $\dot{s}>0$, making s(t) increase over time. The resulting control error is then computed with:

(22) $$\begin{array}{c} e_{q}\left(t\right)=q^{d}\left(s\left(t\right)\right)=q\left(t\right)\\ e_{p}\left(t\right)=p_{x}^{d}\left(s\left(t\right)\right)-p_{x}\left(t\right) \end{array}$$

The controller $\ddot{q}$ would firstly be designed as:

(23) $$\ddot{q}={\ddot{q}}^{d}+k_{p}{\ddot{e}}_{q}\left(t\right)=\frac{dq^{d}\left(s\left(t\right)\right)}{ds}\ddot{s}+\frac{d^{2}q^{d}\left(s\left(t\right)\right)}{ds^{2}}{\dot{s}}^{2}+k_{p}{\ddot{e}}_{q}\left(t\right)=\frac{dq^{d}\left(s\left(t\right)\right)}{ds}\ddot{s}+\pi \left(s,\dot{s},q,\dot{q}\right)$$

Let the angular momentum dynamics mentioned in Equation (15) be with $p_{x}\left(t\right)=p_{x}^{d}\left(s\left(t\right)\right)$, then we can have:

(24) $$\left(W_{0}\left(q\right)+p_{x}^{d}\left(s\right)W_{p}\left(q\right)\right)\left(\frac{dq^{d}\left(s\left(t\right)\right)}{ds}\ddot{s}+\pi \right)+l_{0}\left(q,\dot{q}\right)+p_{x}^{d}\left(s\right)l_{p}\left(q,\dot{q}\right)=0$$

And with Equation (9), we can know τ as:

(25) $$H\left(q\right)\left(\frac{dq^{d}\left(s\left(t\right)\right)}{ds}\right)\ddot{s}+\pi +C\left(q,\dot{q}\right)=B\left(q,\dot{q}\right)\tau +\Psi \left(q\right){\left(q\right)}^{T}F$$

Finally, the designed controller with $\ddot{s}$ and τ can ensure the dynamical balance of the humanoid robot system, and can be represented as:

(26) $$\begin{array}{c} \ddot{s}=\frac{\left(W_{0}\left(q\right)+p_{x}^{d}\left(s\right)W_{p}\left(q\right)\right)\pi -l_{0}\left(q,\dot{q}\right)-p_{x}^{d}\left(s\right)l_{p}\left(q,\dot{q}\right)}{\left(W_{0}\left(q\right)+p_{x}^{d}\left(s\right)W_{p}\left(q\right)\right)\frac{q^{d}\left(s\right)}{ds}}\\ \tau =B_{a}^{-1}\left(q,\dot{q}\right)\left(H_{a}\left(q\right)\left(\frac{dq^{d}\left(s\left(t\right)\right)}{ds}\ddot{s}+\pi \right)+C_{a}\left(q,\dot{q}\right)-\Psi_{a}\left(q\right)F\right) \end{array}$$

## 4. Simulation and Experiment

### 4.1. Hardware Platform

**Figure 4:**
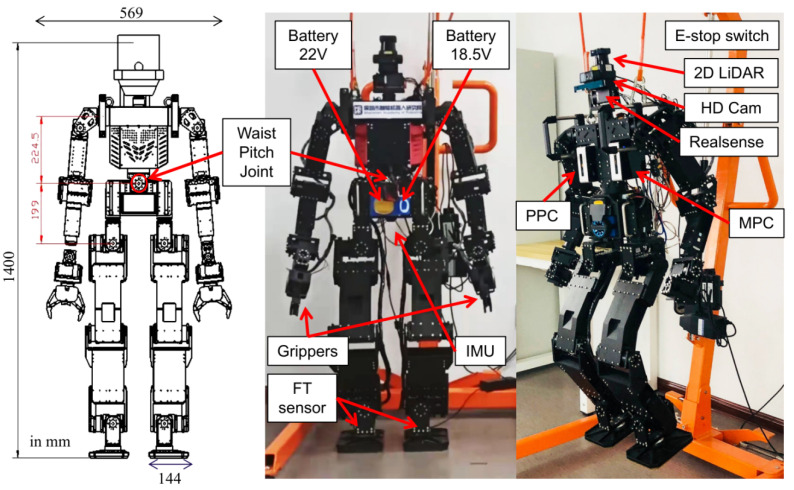
The full-size humanoid robot hardware. (**Left**): design paper with size; (**Middle**): real robot with shell; (**Right**): real robot without shell.

Our humanoid robot which is shown in Figure 4, developed at the Shenzhen Academy of Robotics (SZAR), weighs 44 kg and features two 6-DOF legs, 6-DOF arms, a 2-DOF head, and a 2-DOF waist. It uses 29 ROBOTIS brushless motors powered by a 22 V battery, with integrated sensors for precise control. Two six-axis force/torque sensors at the ankles and an IMU at the body’s center provide data for ZMP and dynamic state estimation. The robot’s hardware is similar to the THORMANG3 model. The system is powered by two onboard Intel^®^ NUC computers (i5, 8 GB RAM, 128 GB SSD). One handles motion control at 100 Hz (MPC), while the other handles perception tasks (PPC) with SLAM, using LiDAR and cameras. Both wirelessly connect to an external operation PC for user interface and monitoring, all running Ubuntu 18.04 with ROS Melodic.

### 4.2. Simulation of Biped Robot Locomotion Under Disturbances

**Figure 5:**
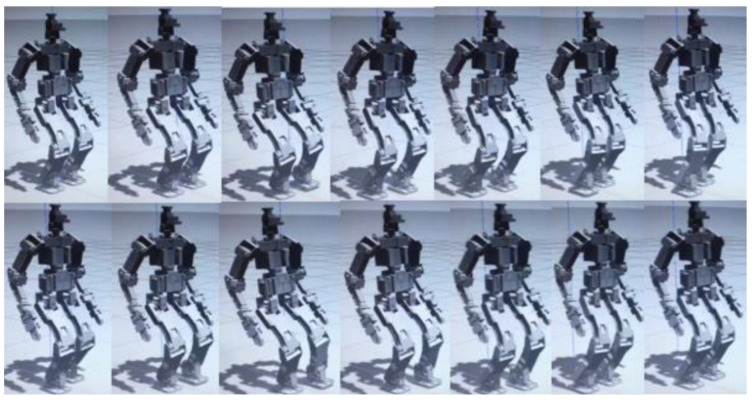
Simulation test a (0.1 m, 1.0 s, 4 steps forward) without external disturbance, with the simplified dynamic model and IMU, F/T sensor noise.

To initially validate the proposed method under ideal conditions, a dynamic simulation was conducted in Gazebo, free from disturbances and sensor noise. The target locomotion parameters were set to 0.1 m step length, a cycle time of 1.0 s, and four forward steps, as depicted in Figure 5. In the absence of external disturbances, the perceptive framework successfully generated a smooth Zero Moment Point (ZMP) trajectory, along with corresponding foot placement and center of gravity (CoG) paths, as illustrated in Figure 5.

**Figure 6:**
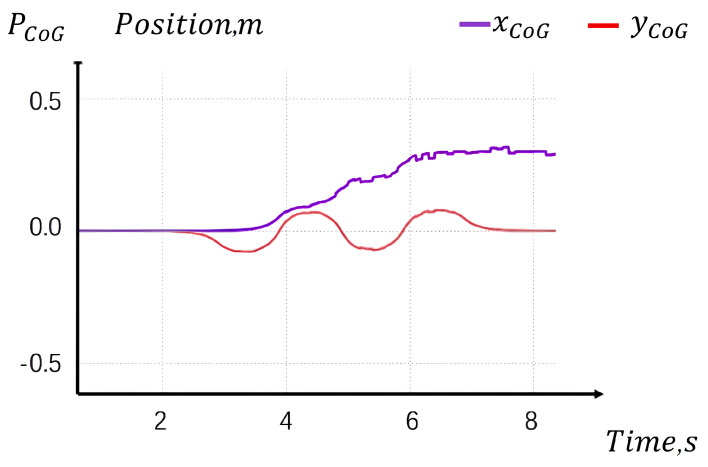
Simulation test result of a: The reference CoG position without any disturbance.

As shown in Figure 6, the overall Center of Mass (CoM) tracking performance closely matches that of the time-based framework. The locomotion process was completed within the expected duration, approximately 4 s for simulation (a).

**Figure 7:**
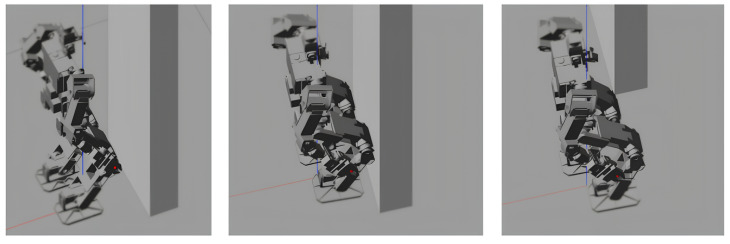
Simulation test b (0.1 m, 1.0 s, 4 steps forward) with additional external disturbance.

Subsequently, an unexpected obstacle was introduced, temporarily blocking the robot’s right foot (depicted within the red rectangle in Figure 7). Specifically, a static, wall-like obstacle impeded the right foot for 0.2 s. This can be interpreted as an external force being applied in the opposite direction along the *x*-axis for 0.2 s.

In Figure 5, obtained from simulation (a), the robot demonstrates a distinct yet stable gait over different time intervals (please refer to the attached video for further explanation). This variation is reflected in the time-dependent displacement s(t), influenced by uncertainties in the Linear Inverted Pendulum (LIP) model, control errors, and disturbances originating from both the ground and the robot itself.

**Figure 8:**
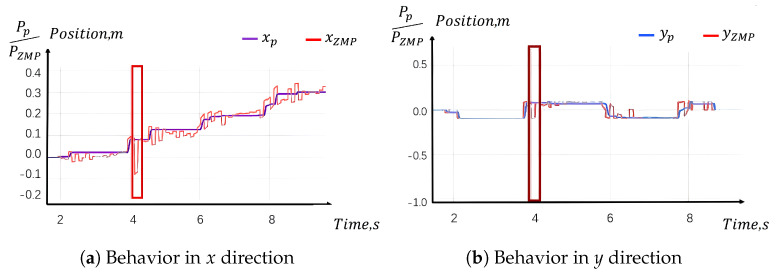
Simulation test b result: The ZMP and projected ZMP with an unexpected disturbance.

In simulation test (b), a temporary blocking force is applied to the right knee while the robot is in the leg-swinging phase. As illustrated in Figure 8, this external force significantly impacts the actual Zero Moment Point (ZMP) values along both the x and y axes, deviating from the desired path. However, the perceptive planner is capable of pausing temporarily, as it maintains its reference value at the moment the force is applied. Consequently, the desired right foot pose is also paused, along with the center of gravity (CoG) and the left foot. The additional time created by this pause is effectively used by the ZMP ankle and pelvis controller to correct the ZMP deviations. As a result, the remainder of the planned gait is executed successfully without the need for re-planning, as shown in Figure 8. This demonstrates a key feature of the system, where the reference “waits” until the control error is minimized.

**Figure 9:**
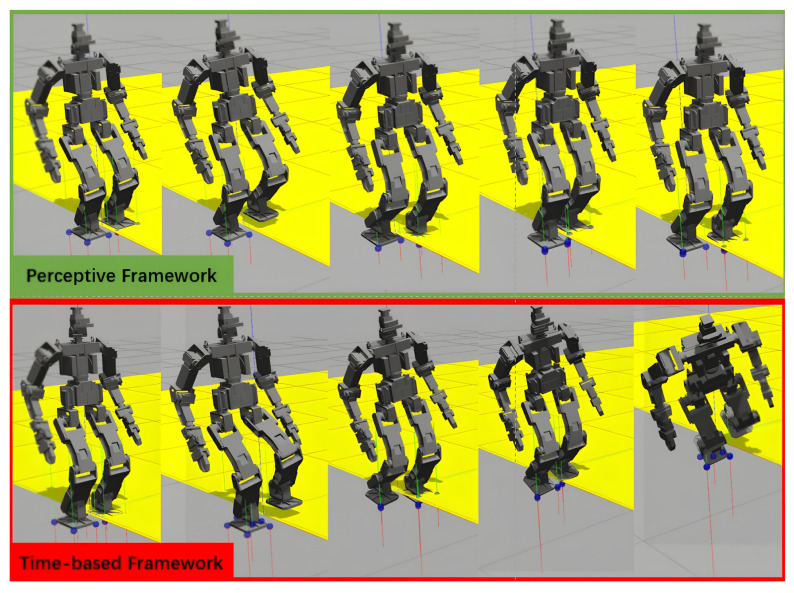
Simulation test c (0.1 m, 1.0 s, 3 steps forward) with the hard ground in gray and the soft and elastic ground in yellow as an unexpected disturbance.

To clearly demonstrate the robustness and advantages of the proposed method, we evaluate its performance on soft or elastic surfaces, such as desert terrain or artificial elastic materials. These surfaces require significantly greater deformation or depth along the z axis to generate the desired ground reaction force. However, traditional robot perception algorithms struggle to detect such soft or elastic surfaces in advance, as they appear similar to normal ground in both 2D and 3D before contact is made. To create a more challenging scenario, we modified half of the ground’s elastic properties in the Gazebo simulation (with a stiffness parameter of kp = 500, resembling the elasticity of an inflated yoga ball), as illustrated in yellow in Figure 9. The gray areas in Figure 9 represent hard, rigid surfaces, comparable to standard concrete (with a stiffness parameter of kp = 500,000).

**Figure 10:**
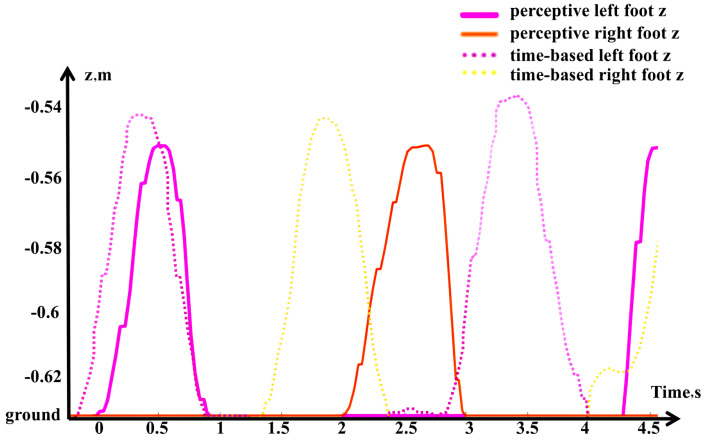
Simulation test c result: Walking on soft/elastic ground (yellow) simulation with feet z axis position comparison between perceptive framework and time-based framework.

In the force/position hybrid control of the left leg, the position control error increases unexpectedly in order to generate the desired ground reaction force, due to the unanticipated softness of the ground. With the original *z* axis velocity, the left foot requires more time to step deeper compared to the right foot, which is on the rigid gray surface. As shown in the lower row of snapshots in Figure 9, the time-based control triggered the second step according to the global clock, but prematurely. This behavior is confirmed in Figure 10, where the first right foot swing occurs approximately 0.5 s later, and the second left foot swing about 1.5 s later in the perceptive framework compared to the time-based approach. In contrast, the perceptive framework demonstrates adaptability and robustness in this test, successfully completing the locomotion task.

### 4.3. Experimental Test of Biped Robot Under Unexpected Disturbances

**Figure 11:**
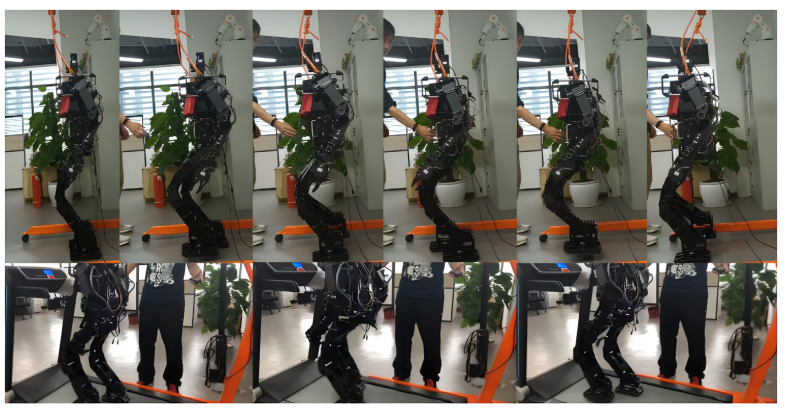
Locomotion with external disturbance and max-speed experiments on real full-size humanoid.

To evaluate the method under more realistic conditions, locomotion experiments were performed using a full-size humanoid robot on a standard treadmill, as shown in Figure 11. After several trials, the proposed method enabled the robot to achieve fast forward walking at a linear speed of up to 0.42 m/s while maintaining dynamic balance, further validating the method’s robustness.

## 5. Discussion

A significant challenge in comparing humanoid robot control algorithms lies in the lack of standardized evaluation frameworks. Unlike fields such as computer vision, humanoid robot control is highly hardware-dependent, with algorithms often tailored to specific proprietary platforms, making cross-platform comparisons impractical. Furthermore, the implementation of these algorithms is complex, requiring months of expertise and calibration to reproduce results on different systems. While metrics like ZMP trajectory tracking exist for stable walking, universally applicable benchmarks for dynamic and complex behaviors remain elusive, underscoring the need for shared evaluation criteria in future research.

The simulation and experimental results demonstrated the effectiveness of the perceptive motion planning framework under various conditions, including external disturbances and environmental uncertainties. The simulation results clearly show the system’s robustness when external disturbances, such as temporary foot blockage, are introduced. Specifically, the framework’s ability to pause motion, synchronize corrective actions, and maintain ZMP within the desired trajectory highlights its superiority over traditional time-based control. This robustness can be attributed to the dynamic recalibration of the perceptive reference in real-time. In practice, this feature could significantly enhance the humanoid robot’s performance in unstructured or unpredictable environments, such as disaster response or crowded public spaces.

Following that, we have the test performance when facing the elastic ground. When comparing locomotion on rigid versus elastic surfaces, the perceptive framework demonstrated remarkable adaptability. Unlike traditional control, which heavily relies on precise timing, the perceptive approach adjusted the foot placement and gait cycle dynamically. The delayed initiation of the next step while compensating for the increased depth on the elastic terrain illustrates the flexibility of the framework. This result is significant for applications requiring the traversal of varied terrains, such as exploration or construction robotics.

Lastly, a real-world experiment has been conducted. The experimental validation on a treadmill further confirmed the framework’s real-world viability. The robot achieved a maximum speed of 0.42 m/s while maintaining balance. This success underlines the practical implementation of perceptive motion planning for real-time humanoid control.

The perceptive framework’s ability to outperform time-based counterparts in scenarios with dynamic uncertainties, as evidenced by the simulation and experimental data, provides a compelling argument for its broader adoption. The reduced need for re-planning and the capability to adapt to unexpected conditions without interrupting the task pipeline are particularly noteworthy.

## 6. Conclusions

In conclusion, this paper presents a theoretical analysis and the experimental validation of a perceptive motion reference framework for controlling multiple robotic limbs on a “floating base” system. The proposed method simplifies synchronized motion control across limbs without requiring the temporal coordination of individual movements. It also enhances the system’s robustness and adaptability by quickly adjusting to control errors or external disturbances, maintaining the execution of planned trajectories without the need for re-planning.

While the current study focuses on humanoid platforms and balance criteria, several avenues for future research could expand the capabilities and applications of this framework. First, extending the framework to integrate advanced machine learning algorithms for adaptive motion prediction could further improve performance in highly dynamic and uncertain environments. Second, exploring the integration of tactile sensors and advanced proprioceptive sensing might enhance the robot’s ability to interact more effectively with complex and variable terrains.

Additionally, the framework’s adaptability could be tested on other multi-limbed robots, including quadrupeds and exoskeleton systems, to assess its generalizability. Future works could also investigate the application of this methodology in real-time collaborative tasks involving human–robot interaction, such as industrial assembly lines or medical assistive robotics. Furthermore, optimizing computational efficiency to enable faster decision-making in dense obstacle environments remains a critical area for development.

Finally, the integration of this perceptive motion reference framework with external object manipulation and coordinated multi-robot systems opens up exciting possibilities in logistics, search-and-rescue operations, and extraterrestrial exploration.

By addressing these challenges and opportunities, the proposed framework has the potential to significantly advance the state-of-the-art in humanoid robot motion planning and control.

## Data Availability

The original contributions presented in this study are included in the article. Further inquiries can be directed to the corresponding author.
